# The Application of an Intelligent *Agaricus bisporus*-Harvesting Device Based on FES-YOLOv5s

**DOI:** 10.3390/s25020519

**Published:** 2025-01-17

**Authors:** Hao Ma, Yulong Ding, Hongwei Cui, Jiangtao Ji, Xin Jin, Tianhang Ding, Jiaoling Wang

**Affiliations:** 1 College of Agricultural Equipment Engineering, Henan University of Science and Technology, Luoyang 471003, China; ding18437910053@163.com (Y.D.);; 2 Key Laboratory of Modern Agricultural Equipment, Ministry of Agriculture and Rural Affairs, Nanjing Institute of Agricultural Mechanization, Nanjing 210014, China; wangjiaoling@caas.cn; 3 Key Laboratory of Intelligent Equipment and Robotics for Agriculture of Zhejiang Province, College of Biosystems Engineering and Food Science, Zhejiang University, Hangzhou 310058, China

**Keywords:** *Agaricus bisporus*, harvesting device, motor control algorithm, machine vision, low damage

## Abstract

To address several challenges, including low efficiency, significant damage, and high costs, associated with the manual harvesting of *Agaricus bisporus*, in this study, a machine vision-based intelligent harvesting device was designed according to its agronomic characteristics and morphological features. This device mainly comprised a frame, camera, truss-type robotic arm, flexible manipulator, and control system. The FES-YOLOv5s deep learning target detection model was used to accurately identify and locate *Agaricus bisporus*. The harvesting control system, using a Jetson Orin Nano as the main controller, adopted an S-curve acceleration and deceleration motor control algorithm. This algorithm controlled the robotic arm and the flexible manipulator to harvest *Agaricus bisporus* based on the identification and positioning results. To confirm the impact of vibration on the harvesting process, a stepper motor drive test was conducted using both trapezoidal and S-curve acceleration and deceleration motor control algorithms. The test results showed that the S-curve acceleration and deceleration motor control algorithm exhibited excellent performance in vibration reduction and repeat positioning accuracy. The recognition efficiency and harvesting effectiveness of the intelligent harvesting device were tested using recognition accuracy, harvesting success rate, and damage rate as evaluation metrics. The results showed that the *Agaricus bisporus* recognition algorithm achieved an average recognition accuracy of 96.72%, with an average missed detection rate of 2.13% and a false detection rate of 1.72%. The harvesting success rate of the intelligent harvesting device was 94.95%, with an average damage rate of 2.67% and an average harvesting yield rate of 87.38%. These results meet the requirements for the intelligent harvesting of *Agaricus bisporus* and provide insight into the development of intelligent harvesting robots in the industrial production of *Agaricus bisporus*.

## 1. Introduction

The *Agaricus bisporus* (J. E. Lange) Imbach is a low-calorie, high-dietary-fiber, and nutrient-rich food that is highly consumed, playing an important role in sustainable agriculture [1,2], and *Agaricus bisporus* is an edible mushroom cultivated in more countries and across larger areas worldwide [3]. It can be processed into a variety of food products and medicines and has high economic value [4]. Therefore, there is a high demand for *Agaricus bisporus* in the market. The high demand for button mushrooms has prompted the industrialization and automation of *Agaricus bisporus* mushroom cultivation. Consequently, most production processes of button mushrooms are currently automated. However, button mushrooms are still manually harvested, leading to low production efficiency, high damage rates, and poor quality, which hinder their industrial development [5,6]. Therefore, developing a mushroom harvesting robot is an urgent task. Achieving automated harvesting can eliminate labor, reduce production costs, and enhance market competitiveness, which are of great significance to the industry’s advancement [7,8].

Due to the multiple layers of *Agaricus bisporus* mushroom beds arranged in parallel and the narrow space in a mezzanine, a picking device needs to be designed according to the environment of the mushroom room and the required function, which requires the equipment to have a simple structure, high efficient picking speed, reliable stability, smaller size, flexible end-effector, and other structures [9,10]. Therefore, the overall design of the structure of *Agaricus bisporus*-picking machinery is difficult. In order to meet these requirements, more researchers are carrying out extensive research. The *Agaricus bisporus* mushroom-picking robot designed by Reed [11] had three main components: a vision system for *Agaricus bisporus* mushroom localization image analysis, a control algorithm for controlling a Cartesian robot, and a dedicated *Agaricus bisporus* mushroom-picking end-effector, but the tested recognition accuracy was 84%, and the successful picking rate was only 57%. In order to improve the picking success rate, Recchia [12] proposed the use of a robotic arm, which was a six-degree-of-freedom collaborative robot designed to adapt to the needs of mushroom picking at different angles and positions for the realizing of complex picking movements, but it was not suitable for operation in a mezzanine space. Therefore, more researchers choose to use a wheeled *Agaricus bisporus* mushroom-picking robot. Hu [13] designed an *Agaricus bisporus* mushroom-picking robot, which was composed of an outer frame, a Cartesian robotic arm, a camera, an electrical box, a chassis, wheels, etc., and used the visual area, picking area, and auxiliary area to cooperate with other such robots to complete the identification and picking of *Agaricus bisporus* mushrooms. Yang et al. [14], based on the above-mentioned vehicle-mounted picking device, designed a new flexible end-effector using vacuum negative pressure, which effectively reduced the damage caused by the picking process. The robot was tested and was found to achieve a picking success rate of 88.2% and a picking damage rate of 2.9%. Zhong et al. [15] designed an *Agaricus bisporus* mushroom-picking device composed of a depth camera, a picking module, a retractable end-effector, an active wheel, and a follower wheel; the device captured images through the camera, and the recognition algorithm detected and located *Agaricus bisporus* mushrooms in the images. The picking process was completed using the picking module and the end-effector. Although the wheeled *Agaricus bisporus*-picking robot can complete the positioning and picking of *Agaricus bisporus* in a narrow space, it comes into direct contact with the mushrooms at the edge of the mushroom bed during travel, which results in damage to these mushrooms, such as extrusion, scraping, and breakage. Wang et al. [16] designed a picking robot suitable for the operation of multilayer standard mushroom beds; its main mechanism contains a mobile lifting platform, a telescopic track extension platform, and a SCARA picking robotic arm. The robot features aisle extension, flexible movement, a high picking speed, and a lightweight design. A news report describes a mobile humanoid *Agaricus bisporus* mushroom-picking robot [17]. The picking device featured a side-picking mechanism, which could rise to the appropriate position based on the height of the mushroom layer and pick mushrooms using two SCARA picking arms. Although the side-picking device can reduce damage to *Agaricus bisporus* mushrooms at the edge of the mushroom bed, the lifting platform of this machine raises its center of gravity, making it prone to tipping over when elevated. Additionally, the stability of the SCARA picking arm during the picking process needs further investigation. The above-mentioned Agaricus bisporus-picking devices have problems such as a large size, complex mechanical structures, great damage to Agaricus bisporus, and low picking stability.

Aiming to address issues such as the complex mechanical structure of *Agaricus bisporus*-picking devices, high picking damage rates, and poor picking stability, this study proposes an intelligent *Agaricus bisporus*-picking device based on machine vision. The study aims to (1) design a device with a simple structure and high picking efficiency that can operate in narrow spaces, using the FES-YOLOv5s model to achieve accurate identification of densely packed *Agaricus bisporus*; and (2) implement a control algorithm to enable the picking device to automatically, quickly, and stably pick *Agaricus bisporus*. To this end, the following research was carried out: (1) a gantry-type *Agaricus bisporus*-picking device was designed according to the *Agaricus bisporus* growing environment and the height of the mushroom beds, and walking rails were set up between the mushroom layers; and (2) the S-type accelerating and decelerating motor driving algorithm was adopted to minimize vibration impact during the picking process. The stability and scientific validity of the picking device were verified through experiments on the motor control algorithm and the picking process.

## 2. Materials and Methods

### 2.1. Climbing Mechanism

**Figure 1:**
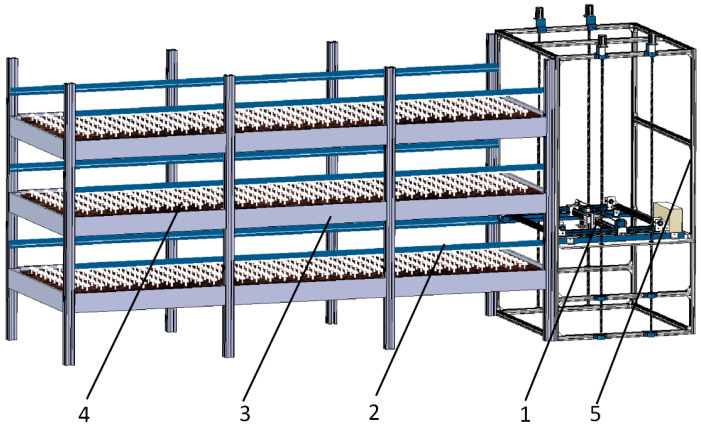
Structural diagram of the *Agaricus bisporus*-harvesting platform: 1. harvesting robot; 2. U-shaped guide rail; 3. mushroom rack; 4. mushroom bed; 5. climbing device.

The *Agaricus bisporus*-harvesting platform mainly comprises a climbing device and an intelligent *Agaricus bisporus*-harvesting device. The overall structure is shown in Figure 1. The climbing device comprises a support frame, a climbing platform, a lead screw, and other components. It transports the *Agaricus bisporus*-harvesting device to different layers of the mushroom racks to complete the harvesting tasks and to transport the fully loaded harvesting robot to a designated location for the replacement of the *Agaricus bisporus* collection frame. The lead screw is connected to the climbing platform, and the stepper motor drives the lead screw’s movement, enabling the climbing platform to move the harvesting robot up and down. Once the designated height is reached, the harvesting robot advances into the corresponding layer of the mushroom rack to perform harvesting tasks.

### 2.2. Structure of the Intelligent Harvesting Device

**Figure 2:**
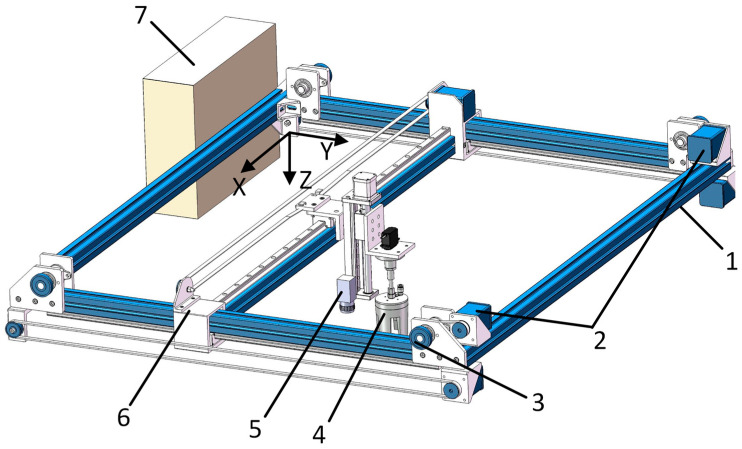
Structural diagram of the intelligent *Agaricus bisporu*-harvesting device: 1. frame; 2. frame stepper motor; 3. track wheel; 4. flexible manipulator; 5. camera; 6. gantry-style robotic arm; 7. control system.

The intelligent *Agaricus bisporus*-harvesting device was designed according to the structure of the *Agaricus bisporus* substrate bed, which has an overall rectangular shape. It mainly comprises a frame, a camera, a gantry-style robotic arm, a flexible manipulator, and a control system (Figure 2). The device uses a camera to identify and locate *Agaricus bisporus*, and its control system coordinates the gantry-style robotic arm and flexible manipulator to achieve the flexible harvesting of *Agaricus bisporus*. This approach reduces the damage rate during harvesting while improving operational efficiency.

### 2.3. Working Principle

**Figure 3:**
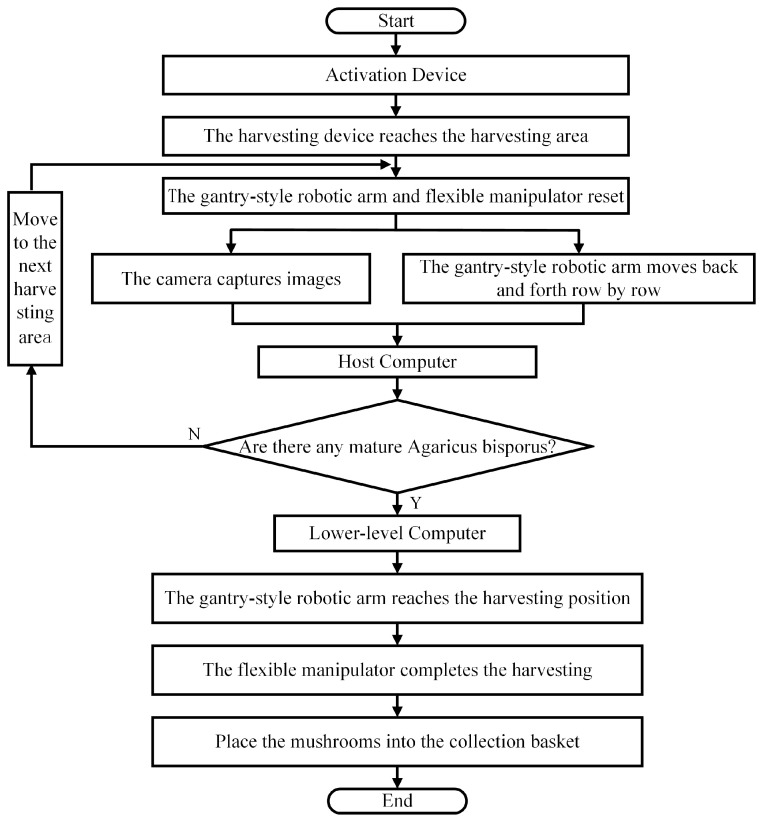
Workflow diagram of the *Agaricus bisporus*-harvesting device.

The climbing device is located on one side of the mushroom rack. It can transport the intelligent *Agaricus bisporus*-harvesting device to a mushroom bed that is ready for harvesting. A U-shaped track is installed on the mushroom rack, enabling the harvesting device to move along the track. Once it reaches the harvesting area, the harvesting operation begins. The internal gantry-style robotic arm can move freely along the X, Y, and Z axes, covering the entire space within the frame. When the harvesting device moves into the harvestable area, the gantry-style robotic arm and flexible manipulator reset to their initial positions. The camera, mounted on the lead screw sliding platform of the gantry-style robotic arm, follows the arm in a reciprocating motion, scanning row by row. The collected information is transmitted to the host computer. The host computer processes the image data, identifies and locates mature *Agaricus bisporus*, and generates control commands to be sent to the lower-level computer. After receiving the command, the lower-level computer controls the gantry-style robotic arm to move to the harvesting position and simultaneously controls the flexible manipulator to complete the *Agaricus bisporus* harvesting, placing it in the collection basket. The working process of the *Agaricus bisporus*-harvesting device is shown in Figure 3.

### 2.4. Key Component Design

#### 2.4.1. Structure of the Gantry-Style Robotic Arm

Because *Agaricus bisporus* is cultivated on multilayered, parallel-arranged mushroom beds with limited space between each layer, operating large harvesting devices within the cultivation racks becomes challenging.

**Figure 4:**
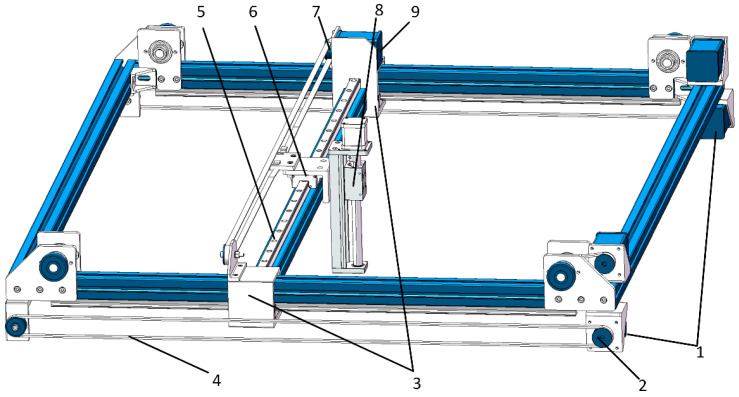
Truss-type mechanical arm structure diagram. 1. Y-axis stepper motor; 2. synchronous pulley; 3. Y-axis sliding module; 4. timing belt; 5. beam; 6. X-axis sliding module; 7. synchronous pulley; 8. X-axis stepper motor; 9. lead screw sliding platform.

As shown in Figure 4, the structure of the gantry-style robotic arm mainly comprises a beam, a lead screw sliding platform, synchronous pulleys, timing belts, an X-axis stepper motor, an X-axis sliding module, a Y-axis stepper motor, and a Y-axis sliding module. It controls the movement of the flexible manipulator within the harvesting area to complete the harvesting operation. Two Y-axis stepper motors are located at the lower ends of the wide sides of the frame and are connected to the synchronous pulleys. The two Y-axis sliding modules are installed on both sides of the wide edges of the frame, which are connected to the beam on the inside and the timing belt on the outside. The Y-axis stepper motor drives the synchronous pulleys and timing belts, guiding the beam to move along the Y-axis within the harvesting area. The lead screw sliding platform is vertically installed above the beam and is connected to the X-axis sliding module. It is driven by the X-axis stepper motor, which rotates the synchronous pulleys and timing belts, causing the lead screw sliding platform to move along the X-axis within the harvesting area. The lead screw sliding platform is connected to the flexible manipulator through a connector. The rotation of the stepper motor drives the flexible manipulator to move up and down along the Z-axis. The three-axis stepper motors collaboratively control the flexible manipulator to move flexibly within three-dimensional space, thereby enabling the precise harvesting of *Agaricus bisporus*.

#### 2.4.2. Structure of the Flexible Manipulator

**Figure 5:**
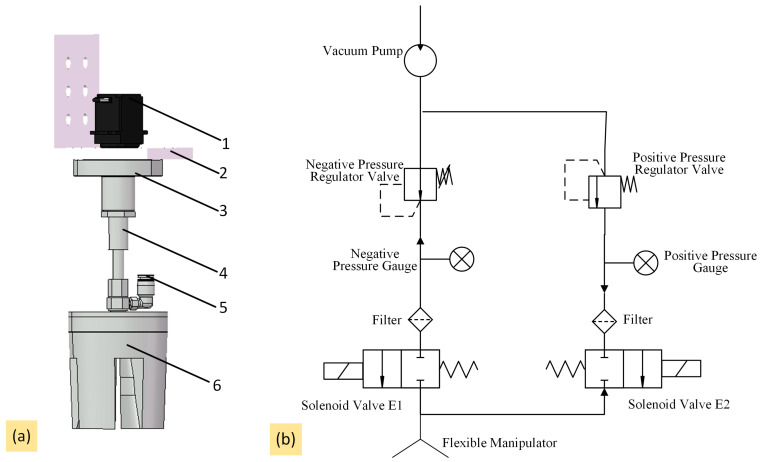
Manipulator structure and pneumatic driving diagram: (**a**) flexible manipulator structure diagram; (**b**) pneumatic drive circuit. 1. Servo motor; 2. connector; 3. spring flange; 4. telescopic rod; 5. air inlet; 6. pneumatic flexible fingers.

Due to the susceptibility of the fruiting body of *Agaricus bisporus* to damage and the direct interaction of the manipulator with the mushroom during harvesting, the harvesting manipulator must possess both flexibility and rigidity [18]. Additionally, it must maintain a constant contact force to achieve the stable harvesting of *Agaricus bisporus* [19]. To meet these requirements, a pneumatic, flexible clamping-type manipulator was selected in this study to achieve the low-damage and stable harvesting of *Agaricus bisporus*. The mechanical structure of the flexible manipulator is shown in Figure 5a.

The flexible manipulator mainly comprises a servo motor, spring flange, telescopic rod, air inlet, and pneumatic flexible fingers. The servo motor is connected to the spring flange through a connector. When the servo motor rotates, it drives the flexible fingers to rotate, thus separating the mushroom stem from the substrate. This separation reduces the amount of substrate lifted during the harvest and ensures that other mushrooms in the substrate continue to grow. The other end of the spring flange is connected to the pneumatic flexible fingers through a telescopic rod, enhancing the adaptability of the flexible manipulator along the Z-axis. Additionally, owing to the consistent growth height of mature *Agaricus bisporus*, the descent height of the manipulator was set to a fixed value to minimize damage to the mushrooms during the descent of the flexible fingers. Due to the susceptibility of the mushroom cap of *Agaricus bisporus* to damage during harvesting, the pneumatic flexible fingers were designed based on mimicry of soft beak structures. The four fingers were made of flexible material. During the harvesting process, they exert pressure around the edges of the mushroom cap, reducing the pressure on the cap and minimizing mechanical damage.

The pneumatic flexible manipulator requires precise pneumatic pressure control [20], and the pneumatic drive circuit used is shown in Figure 5b. As the flexible manipulator requires both positive and negative pressure control, a dual-purpose vacuum pump that can both evacuate and inflate was selected. To effectively control the flexible manipulator by adjusting the generated negative pressure, a negative pressure regulator valve was selected, with an adjustment range of −100 to 1.3 kPa. The vacuum pump is connected to both evacuation and inflation circuits, with the active circuit being controlled by a solenoid valve to operate the harvesting manipulator. In practical tests, the selected pneumatic components were fully sealed with no leakage, and the entire circuit was lightweight and simple, meeting the control requirements.

### 2.5. Control System Design

#### 2.5.1. Control System Design and Working Principle

**Figure 6:**
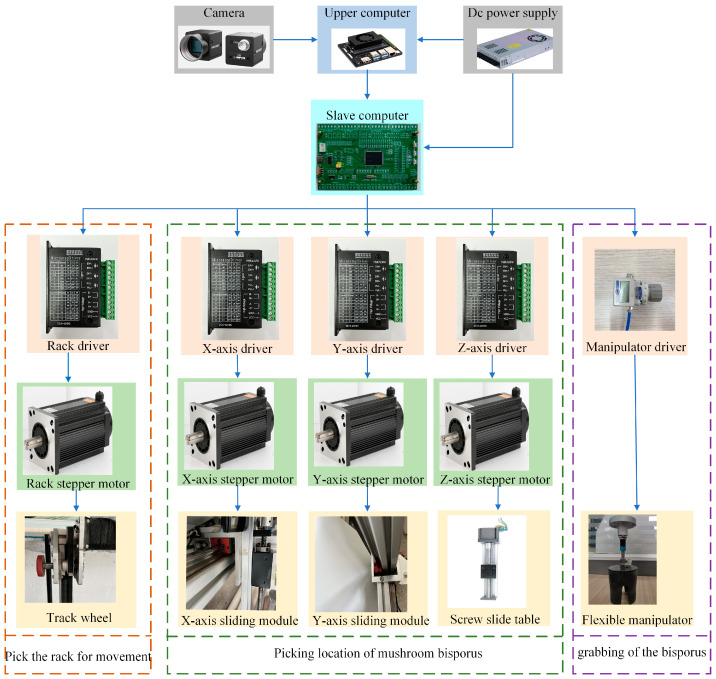
Hardware system of the *Agaricus bisporus*-harvesting device.

The harvesting control system (Figure 6), as the control mechanism of the harvesting device, is mainly responsible for receiving external commands, executing predetermined actions, and providing feedback on the execution of commands. The hardware of the harvesting control system mainly comprises an upper-level computer (Jetson Orin Nano, NVIDIA Corporation, Santa Clara, CA, USA), a lower-level computer (STM32F407, STMicroelectronics, Agrate Brianza, Italy), stepper motor drivers (TB6600, Toshiba Corporation, Tokyo, Japan), and a vacuum pressure regulator valve (IRV10-C06, SMC Corporation, Chiyoda-ku, Japan). The working principle of the harvesting control system is as follows: When the upper-level computer sends a scanning command, the lower-level computer receives and interprets the command, controlling the robotic arm to operate according to the instructions. The camera, mounted on the gantry-style robotic arm, scans and transmits the image data to the upper-level computer. The upper-level computer processes the images, identifies and locates the coordinates of *Agaricus bisporus*, and then transmits the coordinate information to the lower-level computer. After the lower-level computer receives the coordinate information, it directly controls the Y-axis and X-axis stepper motors to position the manipulator above the *Agaricus bisporus*. The motor on the lead screw sliding platform rotates, moving the manipulator downward and controlling it so that it opens. When the lead screw sliding platform descends to the predetermined height, the manipulator closes to grasp the mushroom cap. Then, the servo motor rotates at a certain angle, breaking the mushroom stem, and the flexible manipulator lifts, thereby completing the harvesting of *Agaricus bisporus*. Finally, the X-axis and Y-axis stepper motors, in coordination with the vacuum pressure regulator valve, place the harvested *Agaricus bisporus* into the collection basket, thus completing the harvesting process.

#### 2.5.2. Upper-Level Computer Interface Design

**Figure 7:**
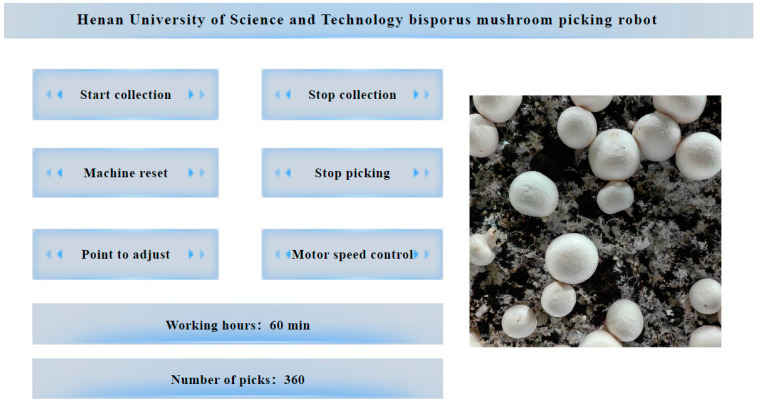
Upper computer page diagram. The (**left**) side of the diagram shows the unit’s function buttons, operating hours, and total amount of picking. On the (**right**) side is the real-time detection screen for *Agaricus bisporus*.

The upper-level computer interface for the *Agaricus bisporus*-harvesting control system is shown in Figure 7 and was designed using QT. The upper-level computer interface mainly controls image acquisition, invokes the *Agaricus bisporus* recognition and localization model, and sends commands to the lower-level computer. The interface mainly comprises an image display module and a button module. The button module includes four functions: start collection, stop collection, start scanning, and start harvesting. The operation steps are as follows: Press “start collection” to turn on the camera, and the image display module will show the camera’s view. Pressing “start collection” initiates the camera’s scanning task along with the gantry-style robotic arm. The image data are transmitted to the upper-level computer, which processes the image and triggers the start harvesting command. Then, the lower-level computer receives the command and begins the harvesting operation.

#### 2.5.3. *Agaricus bisporus* Recognition and Localization Method

(1) Recognition Method of *Agaricus bisporus*

The accurate identification of *Agaricus bisporus* aims to provide essential target information for the robotic arm’s movement. It acts as a critical step in achieving efficient picking and supplies target data for subsequent test trials. *Agaricus bisporus* images were collected from the mushroom cultivation base of Luoyang Songtian Agricultural Development Co. During data collection, the camera was positioned at a height of 380 mm above the mushroom bed, and the images were captured at a resolution of 2448 × 2048 pixels, resulting in a total of 914 images. The original dataset was divided into training, validation, and test sets in a 7:2:1 ratio. To enhance the dataset’s generalization ability and to prevent overfitting, the training and validation sets were augmented through luminance and contrast adjustments, as well as sharpening operations. The final training set consisted of 1311 images, the validation set of 382 images, and the test set of 221 images, with all images annotated using LabelImg [21].

**Figure 8:**
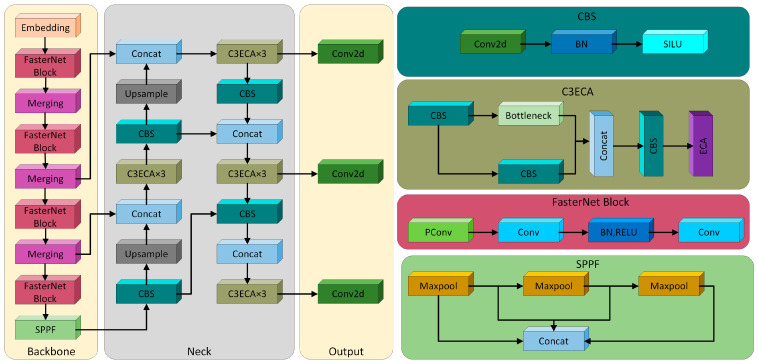
The improved network structure of FES-YOLOv5s. The left side of the figure shows the network structure of FES-YOLOv5s, and the right side shows the network structure of some modules. Adapted from Ma et al. [21].

Due to the dim environment in mushroom rooms and the frequent overlapping and adhesion of *Agaricus bisporus* during the growth period, real-time accurate recognition of *Agaricus bisporus* becomes more challenging [22]. To achieve rapid and accurate recognition of target *Agaricus bisporus*, an improved YOLOv5s-based recognition method has been developed [21]. First, the model uses a FasterNet lightweight network structure to optimize the backbone network of the original model, reducing the complexity of the model and enhancing its computational speed. The SPPF module from the original YOLOv5s was retained to enhance the feature extraction capability of the model. Second, to compensate for the accuracy loss due to the lightweight design, the Efficient Channel Attention (ECA) module was introduced and inserted after the C3 modules in the backbone and neck networks; that is, the ECA module was added to the end of the C3 internal network structure, and the two components were merged to form the C3ECA module. Subsequently, the original C3 module in the backbone and neck network was replaced with the C3ECA module. The introduced module enhances the ability of the model to learn channel interactions, addressing the accuracy decline caused by the lightweight structure. Finally, YOLOv5s was improved using the Soft-non-maximum suppression (NMS) module to suppress candidate boxes, thereby improving the recognition accuracy of the model for occluded and adhered *Agaricus bisporus*. The construction of the FES-YOLOv5s model was thus completed, and its network structure is shown in Figure 8.

The disorderly growth characteristics of *Agaricus bisporus* result in dense distribution and the presence of many overlapping and occluded individuals. The basic NMS module in YOLOv5s has the following characteristic: when two detection boxes are relatively close, the box with the lower score may be mistakenly deleted due to its high overlap with the higher-scoring box, exceeding the deletion threshold. This mistake leads to missed detections during the YOLOv5s detection process, reducing detection accuracy. NMS does not directly delete the lower-scoring detection boxes during candidate box suppression, but it reduces their scores. If the score falls below the suppression threshold, the detection box is deleted. This approach reduces the likelihood of the incorrect suppression of candidate boxes during the suppression phase, thereby improving the detection performance of the model in high-density, adhesion, and occlusion scenarios involving *Agaricus bisporus*.

(2) Localization Method of *Agaricus bisporus*

The accurate positioning of *Agaricus bisporus* ensures that the robotic arm reaches the target precisely, preventing damage to the mushroom caused by improper positioning during the picking process. To accurately obtain the spatial position coordinates of *Agaricus bisporus*, coordinate transformation is needed to convert image coordinates into world coordinates. In the field of computer vision, four common coordinate systems are used: pixel coordinate system, image coordinate system, camera coordinate system, and world coordinate system. The coordinate transformation relationship between the world coordinate system and the depth image coordinate system is shown in Equation (1). To simplify the calculation, the origin of the world coordinate system was set to coincide with that of the camera coordinate system. Therefore, the depth values of the target object are equal in both coordinate systems, i.e., $Z_{c}{=Z}_{w}$, meaning the external parameter matrix of the camera [R T] = [1 0]. The conversion relationship for a point at the same position in both the world coordinate system and the camera coordinate system is shown in Equation (2).

(1) $$Z_{c}\left[\begin{array}{c} u\\ v\\ 1 \end{array}\right]=\left[\begin{array}{c} f_{x}\;\;0\;\;u_{0}\;\;0\\ 0\;\;f_{y}\;\;v_{0}\;\;0\\ 0\;\;\;0\;\;\;1\;\;\;0 \end{array}\right]\left[\begin{array}{c} R\;\;T\\ 0\;\;1 \end{array}\right]\left[\begin{array}{c} X_{w}\\ Y_{w}\\ Z_{w}\\ 1 \end{array}\right]$$

(2) $$\left\{\begin{array}{c} X_{w}=\frac{u-u_{0}}{f_{x}}\times Z_{C}\\ Y_{w}=\frac{v-v_{0}}{f_{y}}\times Z_{C}\\ Z_{w}=Z_{c} \end{array}\right.$$

where $Z_{C}$ is the depth value of the image, mm; u is the X-axis coordinate of any point in the image coordinate system; v is the Y-axis coordinate of any point in the image coordinate system; $u_{0}$ is the X-axis center coordinate of the image; $v_{0}$ is the Y-axis center coordinate of the image; $f_{x}$ is the focal length of the camera along the X-axis; $f_{y}$ is the focal length of the camera along the Y-axis; R is the camera external parameter rotation matrix; T is the camera external parameter translation vector, imm; $X_{w}$ is the X-axis coordinate of any point in the world coordinate system, mm; $Y_{w}$ is the Y-axis coordinate of any point in the world coordinate system, mm; and $Z_{w}$ is the Z-axis coordinate of any point in the world coordinate system, mm.

**Figure 9:**
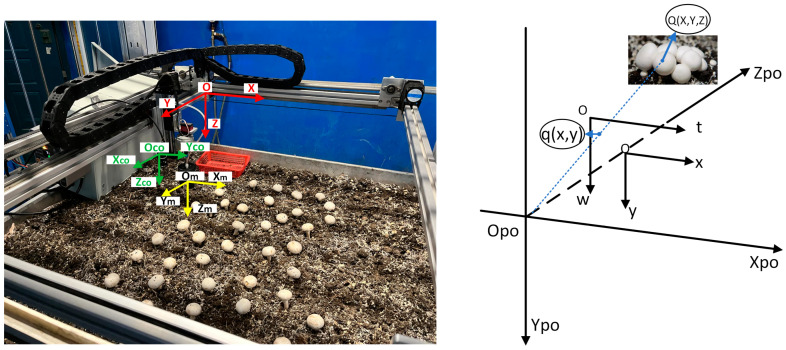
Coordinate relationship diagram: O is the world coordinate system (red coordinate system); Om is the flexible manipulator coordinate system (yellow coordinate system); Opo is the camera coordinate system (green coordinate system); xoy is the image coordinate system; and tow is the pixel coordinate system.

Because the manipulator and the image acquisition device are not in the same coordinate position, a single image may contain multiple *Agaricus bisporus* to be harvested. Coordinate position compensation is required during harvesting to ensure that the manipulator is directly positioned above the mushroom cap of the target *Agaricus bisporus*. As shown in the coordinate relationship in Figure 9, the compensation coordinates can be calculated in the world coordinate system based on the relative position of the harvesting manipulator and the camera, and the formula is as follows:

(3) $$\;\left\{\begin{array}{c} X_{po}=X_{l0}+X_{m}-X_{w\;}\\ Y_{po}=Y_{l0}+Y_{m}-Y_{w} \end{array}\right.$$

where $X_{po}$ is the X-axis coordinate of the position of the gripper at the center of the target *Agaricus bisporus*, mm; $Y_{po}$ is the Y-axis coordinate of the position of the gripper at the center of the target *Agaricus bisporus*, mm; $X_{l0}$ is the X-axis coordinate of the flexible manipulator at the initial point in the world coordinate system, mm; $Y_{l0}$ is the Y-axis coordinate of the flexible manipulator at the initial point in the world coordinate system, mm; $X_{m}$ is the X-axis coordinate of the flexible manipulator’s movement, mm; and $Y_{m}$ is the Y-axis coordinate of the flexible manipulator’s movement, mm.

#### 2.5.4. Control Method for Stepper Motors in Agaricus Bisporus-Picking Robots

(1) Principle of Stepper Motor Control Algorithms

**Figure 10:**
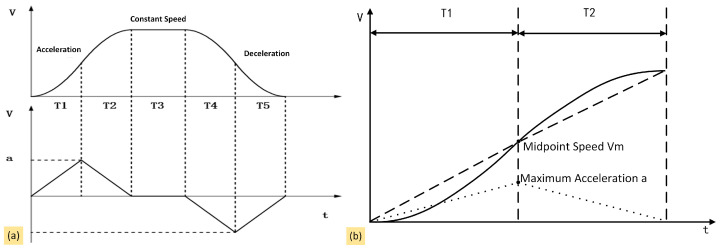
S-type acceleration and deceleration schematic: (**a**) S-type acceleration and deceleration curves; (**b**) acceleration process. T1 and T2 are the acceleration time periods; T3 is the constant speed time period; T4 and T5 are the deceleration time periods; a is the point of maximum acceleration.

The core of *Agaricus bisporus*-picking robot control lies in the cooperative control of multiple stepper motors. Achieving high-speed and stable picking requires increasing motor speed and effectively reducing vibrations during the operational process. A reasonable stepper motor control algorithm serves as the key technical support for achieving stable and efficient picking. In this study, an S-curve acceleration and deceleration motor drive algorithm was used, with the aim of starting at a low speed and gradually accelerating to reduce the shock and vibration caused by sudden changes in acceleration [23]. The S-curve can be divided into three phases: acceleration, constant speed, and deceleration [24]. Based on accuracy, the S-curve acceleration and deceleration algorithm can further be divided into five-phase and seven-phase versions. Because of the high hardware requirements of the seven-phase version, this study adopted the five-phase version. This version is characterized by simplicity, high real-time performance, and low hardware resource consumption (Figure 10a). The acceleration and deceleration processes are symmetrical, resulting in equal acceleration slopes, so T1 = T2 and T4 = T5. Therefore, when selecting the speed, only the T1 and T2 phases need to be calculated, as shown in Figure 10b, where V_0_ is the initial speed, V_M_ is the midpoint speed, V_t_ is the final speed, and a is the acceleration.

(2) Algorithm Implementation

A stepper motor is a type of motor that converts electrical pulse signals into angular or linear displacement. With each input pulse signal, the rotor rotates at a specific angle or advances by one step. The output angular or linear displacement is proportional to the number of input pulses, and the rotational speed is proportional to the pulse frequency [25]. When S-curve acceleration and deceleration control is performed on a stepper motor, the known parameters include the total number of steps, acceleration, deceleration, and maximum speed. The unknowns that need to be calculated are the number of steps in the acceleration and deceleration phases and the next pulse period (i.e., timer count value) [26,27]. The pulse time interval, step angle, position, and speed of a stepper motor are calculated as follows:

(4) $$\delta_{t}=ct_{t}=\frac{c}{f_{t}}$$

(5) $$\alpha =\frac{2\pi }{spr}$$

(6) θ=qα

(7) $$\omega =\frac{\partial f_{t}}{c}=at$$

where $\delta_{t}$ is the pulse time interval, s; c is the timer count value; $t_{t}$ is the timer count period, s; $f_{t}$ is the timer frequency, Hz; α represents the step angle, °; spr is the number of pulses per revolution of the stepper motor; *θ* is the angular position of the stepper motor, °; q is the number of pulses; ω is the angular speed of the stepper motor, °/s; and a is the angular acceleration, °/$s^{2}$.

Using the above formulas, we derived the total time for the first n pulses, the period of the nth pulse, the timer count value for the first pulse, and the timer count value for the nth pulse.

(8) $$t_{n}=\sqrt{\frac{2na}{a}}$$

(9) $$c_{n}t_{n}=t_{n+1}=t_{n}=\sqrt{\frac{2na}{a}}\left(\sqrt{n+1}-\sqrt{n}\right)$$

(10) $$c_{0}=\frac{1}{t_{t}}\sqrt{\frac{2na}{a}}$$

(11) $$c_{n}=c_{n-1}-\frac{2c_{n-1}}{4n+1}$$

For a given total number of steps, acceleration, deceleration, and maximum speed, the corresponding S-curve acceleration and deceleration curve is calculated using these formulas. The speed curve is then sampled at 500 times per second, resulting in a relatively smooth fitted curve. A speed table is generated during the acceleration phase, and the deceleration speed table is obtained by reversing the order of this speed table. After the speed table is generated, the current speed is updated during each timer interrupt based on the pre-calculated speed table. The timer frequency is set to a fixed value during initialization to ensure a constant time interval between timer interrupts. According to the speed values read from the speed table, the motor speed is controlled by adjusting the duty cycle of the pulse-width modulation (PWM) signal. In the STM32, a timer generates the PWM signal, and the timer’s compare register determines the PWM duty cycle. Through an adjustment of the value of this register, the motor speed can be precisely controlled to match the S-curve acceleration and deceleration requirements, thereby achieving precise control of motor movement.

### 2.6. Experimental Section

#### 2.6.1. Experimental Environment

**Figure 11:**
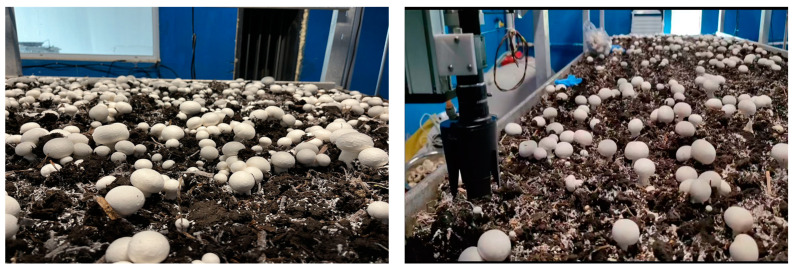
*Agaricus bisporus* mushroom growing room.

To verify the stability and reliability of the *Agaricus bisporus*-harvesting device, experiments were conducted in November 2023 at the *Agaricus bisporus* production laboratory of the College of Agricultural Equipment Engineering, Henan University of Science and Technology. Figure 11 shows the *Agaricus bisporus*-planting laboratory. The laboratory is equipped with variable control devices for cooling, misting, and ventilation. These devices use a multi-factor fuzzy control strategy to regulate environmental temperature, humidity, and carbon dioxide concentration. Additionally, the devices regulate the temperature and humidity of the cultivation substrate. The laboratory has test mushroom racks, each measuring 4.5 m in length and 1.4 m in width. The test materials were selected from *Agaricus bisporus* cultivated in the laboratory. To prevent a large number of deformed or poor-quality mushrooms and to ensure the quality of the *Agaricus bisporus* products, the clusters of mushrooms were thinned, and severely adhered mushrooms were removed after the fruiting bodies emerged. This process resulted in high-quality *Agaricus bisporus* loosely growing on the mushroom beds, ensuring the validity and reliability of the experiment.

#### 2.6.2. Stability Test of the Harvesting Device

In this study, to verify the impact of the S-curve acceleration and deceleration motor drive algorithm on the stability of the harvesting device, stability tests using both the S-curve acceleration and deceleration motor drive algorithm and the trapezoidal acceleration and deceleration motor drive algorithm were conducted. The effects of the two algorithms on the vibration characteristics of the harvesting device were compared and analyzed. During the experiment, a vibration monitoring sensor (WTVB02-485, Shenzhen Weite Intelligent Technology Co., Ltd., Shenzhen, China) was used to collect displacement, speed, and vibration frequency information in the x, y, and z directions. Due to the operation of the harvesting device along three axes, movement along any axis will cause vibrations in the *Z*-axis. To comprehensively capture vibration data during the operation of the harvesting device, the vibration detection sensor was installed on the *Z*-axis stepper motor. In this study, the X, Y, and Z axes of the harvesting device were separately controlled to perform reciprocating movements over their maximum range. When each axis was separately controlled, the vibration monitoring sensor captured the vibration data of the harvesting device in all three directions (x, y, and z), and the vibration information was recorded.

#### 2.6.3. Robotic Arm Repetitive Positioning Test

Because the gantry-style robotic arm is controlled by multiple stepper motors working in coordination, repeated operations and mechanical vibrations can cause cumulative errors, affecting the positioning accuracy of the manipulator. In this study, repetitive positioning tests were conducted to evaluate the repeatability of the gantry-style robotic arm in multiple positioning tasks to test its positioning accuracy. During the experiment, the travel along each axis was divided into short travel (33% of the maximum travel), medium travel (50% of the maximum travel), and long travel (100% of the maximum travel) based on the percentage of the maximum travel. The experiment used a laser displacement sensor (BL-50NZ-485, Boyi Jingke Technology Co., Ltd., Shenzhen, China) to detect the displacement error of the device. However, due to the short range of the laser displacement sensor, the sensor was fixed on the axis to be tested, and the distance was adjusted to display a reading. This reading was recorded as the actual value. Then, the harvesting device was controlled to move along the directions of the *X*, *Y*, or *Z* axis, and the reading from the displacement sensor was recorded as the predicted value. The experiment was conducted using 50 tests for each axis at short, medium, and long travel lengths to evaluate the repetitive positioning accuracy of the harvesting device at different travel lengths.

The root mean square error (*RMSE*) was used to evaluate the accuracy of the single-axis positioning. The RMSE is defined as follows:

(12) $$RMSE=\sqrt{{\frac{1}{N}\sum_{i=1}^{N}\left(y_{i}-\hat{y_{i}}\right)}^{2}}$$

where *RMSE* is the root mean square error, mm; *N* is the total number of tests; $y_{i}$ is the actual value of the i-th test, mm; and $\hat{y_{i}}$ is the predicted value of the i-th test, mm.

#### 2.6.4. Full-Machine Harvesting Test Experiment

(1) Full-Machine Harvesting Test Method

**Figure 12:**
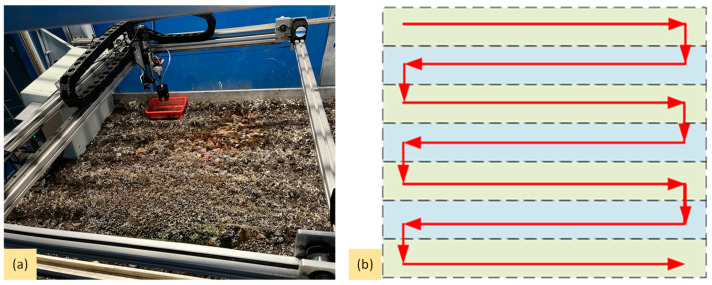
Full system test: (**a**) picking device picking area; (**b**) reciprocating line-by-line detection method.

To verify the comprehensive performance, reasonableness of the design, and practical application effects of the *Agaricus bisporus*-picking robot, a whole-machine test was conducted. This experiment was conducted on the *Agaricus bisporus* mushroom bed in eight selected areas with different growth conditions. Each test area was 1.5 m long, with its width equal to the width of the mushroom rack, corresponding to the working width of the harvesting device. The harvesting areas are shown in Figure 12a. As the total length of each layer of the mushroom rack was 4.5 m, three sets of tests were conducted on each mushroom bed layer, requiring all tests to be completed across different layers of the cultivation rack. The operating path of the harvesting device needs to cover all areas of the mushroom bed to ensure that no *Agaricus bisporus* is missed and to maintain high harvesting efficiency. Therefore, a reciprocal row-by-row scanning method was used in this study to capture all images within each harvesting area and then stitch the images together. The scanning speed of the harvesting device was set to 0.1 m/s, as shown in Figure 12b, with the dashed lines in the figure indicating the range of the captured images.

According to the industry standard NY/T 1790-2009 [28], the *Agaricus bisporus* selected for harvesting must have a diameter ≥35 mm, with an allowable error of 5% (within 2 mm). The total number of *Agaricus bisporus* in each harvesting area that met the harvesting criteria was recorded as *k*. The number of *Agaricus bisporus* that met the criteria and that were successfully recognized by the machine was recorded as *k*_1_. The number of successfully harvested *Agaricus bisporus* was recorded as *k*_2_, and the number of *Agaricus bisporus* with visible damage after harvesting was recorded as *k*_3_. After the experiment, the following metrics were calculated: recognition accuracy *r_de_*, harvesting success rate *r_su_*, *Agaricus bisporus* damage rate *r_da_*, and harvesting yield rate *r_ou_*. The average values of the test results were used.

(2) Evaluation Metrics of Test Results

To evaluate the performance of the *Agaricus bisporus*-harvesting device, the following operational evaluation metrics were used: recognition accuracy *r_de_*, harvesting success rate *r_su_*, severe damage rate *r_da_*, and harvesting yield rate *r_ou_*. Additionally, the missed detection rate *r_md_* and false detection rate *r_fd_* were used to evaluate the recognition accuracy of mature *Agaricus bisporus*.

(13) $$r_{de}=\frac{k_{1}}{k}\times 100\%$$

(14) $$r_{su}=\frac{k_{2}}{k_{1}}\times 100\%$$

(15) $$r_{da}=\frac{k_{3}}{k_{2}}\times 100\%$$

(16) $$r_{ou}=r_{de}\times r_{su}\times \left(1-r_{da}\right)$$

(17) $$r_{md}=\frac{k_{11}}{k}\times 100\%$$

(18) $$r_{fd}=\frac{k_{12}}{k}\times 100\%$$

where $k_{1}$ is the number of *Agaricus bisporus* that met the harvesting criteria and were accurately recognized; k is the total number of *Agaricus bisporus* in the harvesting area that met the harvesting criteria; $k_{2}$ is the number of successfully harvested *Agaricus bisporus*; $k_{3}$ is the number of harvested *Agaricus bisporus* with visible damage; $k_{11}$ is the number of *Agaricus bisporus* that met the harvesting criteria but were missed; $k_{12}$ is the number of *Agaricus bisporus* that met the harvesting criteria but were falsely identified.

## 3. Results

### 3.1. Stability Test Results of the Harvesting Device

The three-axis vibration information of the *Agaricus bisporus*-harvesting device is shown in Table 1, Table 2 and Table 3.

As shown in Table 1, when the device used the trapezoidal acceleration and deceleration motor drive algorithm during *X*-axis movement, the maximum vibration velocity and the maximum vibration displacement in the y-direction were significantly higher than those in other directions, indicating more severe vibration in the y-direction. In contrast, the vibration data for all directions with the S-curve acceleration and deceleration algorithm were lower than those with the trapezoidal algorithm. This phenomenon indicates that the S-curve acceleration and deceleration motor drive algorithm has superior vibration reduction characteristics during *X*-axis movement.

As shown in Table 2, during the *Y*-axis movement with the trapezoidal acceleration and deceleration motor drive algorithm, the vibration velocity and displacement in the y-direction remained high. In addition, the vibration frequency in the z-direction was relatively high. In contrast, when the device used the S-curve acceleration and deceleration motor drive algorithm, the vibrations in the y and z directions were smaller, and the overall vibration level was lower than with the trapezoidal acceleration and deceleration motor drive algorithm.

As shown in Table 3, during *Z*-axis movement, when the device used the trapezoidal acceleration and deceleration motor drive algorithm, the vibration velocity and frequency in the x and y directions were relatively high. In contrast, when using the S-curve acceleration and deceleration motor drive algorithm, the vibration data in all three directions were lower. This observation indicates that the device has better stability and vibration reduction effects during *Z*-axis movement.

The above analysis reveals that using the S-curve acceleration and deceleration motor drive algorithm reduces vibration velocity and displacement during three-axis movement, demonstrating better stability and vibration reduction effects. Therefore, in this study, the S-curve acceleration and deceleration motor drive algorithm was used to control the operation of the *Agaricus bisporus*-harvesting device, ensuring smooth operation and improving operational stability and reliability.

### 3.2. Robotic Arm Repetitive Positioning Test Results

The RMSE results of the three-axis repetitive positioning test are shown in Table 4.

As shown in Table 4, on the X, Y, and Z axes, the RMSE values gradually decrease as the travel distance increases. This phenomenon occurs because during short-distance movements, mechanical gaps and the elastic deformation of the belt prevent the motor rotation from being fully transmitted to the belt, resulting in lower repeat positioning accuracy. As the travel distance increases, the motor rotation is more effectively transmitted to the belt, reducing errors and improving positioning accuracy. In the short-distance positioning accuracy test, the trapezoidal acceleration and deceleration motor drive algorithm exhibited a maximum RMSE of 0.92 mm along the *Y*-axis, while the S-curve acceleration and deceleration motor drive algorithm had a maximum RMSE of 0.91 mm, making it slightly better in short-distance positioning than the trapezoidal acceleration and deceleration motor drive algorithm. In the medium-distance and long-distance positioning accuracy tests, the trapezoidal acceleration and deceleration motor drive algorithm had maximum RMSE values of 0.71 mm and 0.78 mm, respectively, while the S-curve acceleration and deceleration motor drive algorithm had maximum RMSE values of 0.60 mm and 0.68 mm, respectively. These results show that the S-curve algorithm has superior positioning accuracy over the trapezoidal algorithm. This phenomenon occurs because the S-curve acceleration and deceleration motor drive algorithm enables a smoother transition in device speed, reducing shock and vibration, thus improving positioning accuracy. Although the trapezoidal acceleration and deceleration motor drive algorithm also performs well, it is slightly inferior to the S-curve algorithm in terms of dynamic response and stability.

### 3.3. Full-Machine Harvesting Test Results

The results in Table 5 show that the average recognition accuracy for *Agaricus bisporus* targets is 96.72%, indicating that the vision system of the harvesting device effectively recognizes *Agaricus bisporus* and meets the required accuracy. However, in groups 5, 6, and 7, the success rate of *Agaricus bisporus* recognition was below average, indicating a higher incidence of recognition failures and false identifications in these tests. The analysis revealed that these three test groups had a higher planting density of *Agaricus bisporus*, and the substrate was covered with mycelium. The dense growth led to the significant occlusion and overlapping of the mushrooms, which reduced the detection accuracy of the recognition algorithm. Additionally, *Agaricus bisporus* grew on a substrate covered with white mycelium, and substrate clumps resembling the mushrooms in shape could be misidentified, further increasing the false detection rate.

As shown in Table 6, the average harvesting success rate of the *Agaricus bisporus*-harvesting device is 94.95%, with a damage rate of 2.67% and a yield rate of 87.38%. In the seventh test area, the harvesting success rate was the highest at 98.46%, but it did not reach 100% due to the smooth surface and thin film on the mushroom cap. When the flexible manipulator fails to grasp the cap vertically, the *Agaricus bisporus* can easily slip out of the manipulator. Additionally, during the process of delivering the *Agaricus bisporus* to the collection basket, contact with other objects caused the mushrooms to slip, leading to failed grasp. In the eighth test area, the harvesting success rate was the lowest, and the damage rate was the highest, which was significantly higher than the average. This phenomenon occurred because in the eighth area, the mushrooms were densely grown, and the flexible manipulator damaged the surrounding mushrooms while attempting to grasp the target mushroom. Additionally, some mushrooms grew at a certain angle, causing the manipulator to fail in accurately gripping the edge of the mushroom cap and instead grasp other parts, thereby damaging the cap or stem.

## 4. Discussion

In this study, an efficient, accurate, and low-loss *Agaricus bisporus*-picking device was achieved by designing an intelligent picking device based on machine vision. The device utilized the FES-YOLOv5s target detection model combined with a position-compensation-based localization method to ensure the accurate identification and localization of *Agaricus bisporus* mushrooms in a dense growing environment. A truss-type robotic arm structure was designed to enable the flexible movement of the picking device in narrow spaces. To further reduce vibration during the robotic arm’s movement, the S-acceleration algorithm was adopted, significantly improving vibration damping and repetitive positioning accuracy compared with the traditional trapezoidal acceleration and deceleration algorithm.

Compared with existing research on *Agaricus bisporus* picking, the device developed in this study demonstrated significant advantages in picking performance. Yang et al. [14] designed a vacuum-negative-pressure multi-mechanical-arm picking device with a picking success rate of 88.2% and a damage rate of 2.9%. Although the device featured multiple mechanical arms and relatively high picking efficiency, the present device achieved a higher success rate and lower damage rate. Koirala et al. [29] proposed a picking device with a three-finger hybrid gripper jaw, achieving a 100% picking success rate for individual *Agaricus bisporus* mushrooms. However, for clustered mushrooms, the success rate dropped to 64%, and the damage rate reached 36%. In contrast, the pneumatic flexible manipulator in this study demonstrated a lower damage rate and higher picking efficiency. Furthermore, Wang [16] developed a SCARA robotic arm with a picking success rate of 90%, while the truss-type robotic arm in this study, combined with the S-type acceleration and deceleration click-drive algorithm, achieved a success rate of 94.95%. These results indicate that the *Agaricus bisporus*-picking device developed in this study not only improves the stability and accuracy of picking but also effectively reduces damage to *Agaricus bisporus* during the picking process.

Although this study achieved significant results in the field of intelligent picking, some limitations and challenges remain. The single robotic arm design of this device limits picking efficiency in large-scale production scenarios and has room for improvement compared to multi-robotic-arm collaborative systems. Additionally, when dealing with clustered *Agaricus bisporus* mushrooms, the robotic hand’s inability to effectively hold and separate neighboring mushrooms results in a high damage rate. Future research could focus on the following: (1) designing multi-mechanical-arm collaborative systems to enhance efficiency in mass production; (2) developing multi-angle-rotating flexible end-effectors to reduce damage to incline-growing mushrooms; and (3) optimizing vision algorithms to achieve the accurate identification of occluded mushrooms and to plan picking sequences for complex scenarios. Addressing these challenges will enable the intelligent picking device to provide better technical support for the intelligent development of agriculture and promote the edible mushroom industry toward higher levels of factory-based production.

## 5. Conclusions

This study designed and developed an intelligent *Agaricus bisporus* mushroom-picking device based on machine vision. The device consisted of a frame, a camera, a truss-type robotic arm, a flexible manipulator, and a control system. By integrating the FES-YOLOv5s recognition algorithm with a position-compensation-based localization method, the device achieved accurate the recognition and positioning of *Agaricus bisporus* mushrooms in dense cultivation environments. The truss-type robotic arm structure and pneumatic flexible manipulator enabled efficient and low-loss picking. In addition, the application of the S-type acceleration and deceleration algorithm, along with optimized acceleration curves for the stepper motor, reduced the vibration frequencies of the *X*-axis, *Y*-axis, and *Z*-axis to 18.8 Hz, 84.6 Hz, and 42 Hz, respectively. This effectively minimized mechanical shocks and vibrations caused by sudden acceleration changes during harvesting. Compared with the traditional trapezoidal acceleration and deceleration algorithm, the proposed method significantly improved the stability of the robotic arm’s movement and its repeat positioning accuracy. The harvesting test results showed that the device achieved a recognition accuracy of 96.72%, a harvesting success rate of 94.95%, a damage rate of only 2.67%, and an average yield rate of 87.38%. These findings demonstrate that the intelligent harvesting device developed in this study not only improves harvesting efficiency and accuracy but also reduces damage during the harvesting process, providing a feasible and highly efficient solution for factory-based *Agaricus bisporus* mushroom harvesting.

## Figures and Tables

**Table 1:** Picking robot *X*-axis vibration information.

Algorithm	Vibration Direction	Maximum Vibration Velocity (mm·s^−1^)	Maximum Vibration Displacement (µm)	Maximum Vibration Frequency (Hz)
Trapezoidal	x	10	94	170.9
	y	104	4201	0
	z	7	169	10.7
	Average value	40.33	1487	60.23
S-curve	x	6	37	24.5
	y	10	55	4
	z	7	57	27.9
	Average value	7.67	149	18.80

**Table 2:** Picking robot *Y*-axis vibration information.

Algorithm	Vibration Direction	Maximum Vibration Velocity (mm·s^−1^)	Maximum Vibration Displacement (µm)	Maximum Vibration Frequency (Hz)
Trapezoidal	x	25	1273	223.3
	y	43	2198	19.2
	z	2	1162	55.3
	Average value	23.33	1544.33	99.27
S-curve	x	5	43	52.9
	y	7	115	19.2
	z	25	40	12.5
	Average value	12.33	198	84.6

**Table 3:** Picking robot *Z*-axis vibration information.

Algorithm	Vibration Direction	Maximum Vibration Velocity (mm·s^−1^)	Maximum Vibration Displacement (µm)	Maximum Vibration Frequency (Hz)
Trapezoidal	x	6	154	177.1
	y	8	144	102.9
	z	5	73	0
	Average value	6.33	123.67	93.33
S-curve	x	4	39	82.1
	y	4	33	43.9
	z	2	95	0
	Average value	3.33	55.67	42

**Table 4:** Three-axis repetitive positioning test results.

Algorithm	Displacement Axis	Short Distance (mm)	Medium Distance (mm)	Long Distance (mm)	Average Value (mm)
Trapezoidal	*X*-axis	0.46	0.23	0.22	0.30
	*Y*-axis	0.92	0.71	0.70	0.78
	*Z*-axis	0.13	0.13	0.12	0.13
S-curve	*X*-axis	0.42	0.25	0.20	0.29
	*Y*-axis	0.91	0.60	0.68	0.73
	*Z*-axis	0.11	0.12	0.12	0.12

**Table 5:** Detection results of *Agaricus bisporus*.

Serial Number	Recognition Accuracy (%)	Missed Detection Rate (%)	False Detection Rate (%)
1	96.84	1.05	2.10
2	97.17	1.88	0.94
3	97.73	2.27	1.14
4	97.47	1.21	1.32
5	95.54	2.57	1.89
6	96.26	0.00	3.74
7	98.46	1.54	0.00
8	94.31	3.25	2.44
Average value	96.72	2.13	1.72

**Table 6:** Results of analysis of picking robot and manual picking data.

Serial Number	Harvesting Success Rate (%)	Damage Rate (%)	Yield Rate (%)
1	94.74	2.11	89.81
2	94.34	2.83	89.08
3	96.59	1.14	91.13
4	96.2	2.53	82.09
5	92.86	4.46	86.39
6	95.33	1.87	92.69
7	98.46	1.56	81.82
8	91.06	4.88	86.06
Average value	94.95	2.67	87.38

## Data Availability

The data that support the findings of this study are available from the corresponding author upon request.
